# An Efficient Ambulance Service for London

**Published:** 1913-02-22

**Authors:** 


### AN EFFICIENT AMBULANCE SERVICE FOR LONDON.

### Not Politics but Business.

We regret to see that the provision of an efficient ambulance service for London is being made a party question by both sides in the election of members of the London County Council now proceeding. It is a monstrous thing for either the Progressives or the Municipal Reformers to treat this important matter as a party question at all. It is in fact a matter of business, and neither party has done its duty to the ratepayers in regard to it. We showed in our article on the 8th inst., page 565, that of the elected members of the London County Council a clear majority of sixty-three, of whom thirty-three are Municipal Reformers and thirty Progressives, declared themselves in favour of the County Council establishing an efficient ambulance service in London. Of these sixty-three only ten qualified their approval. We gave the names and constituencies of these candidates. It is therefore unwarrantable in our judgment, seeing that both Municipal Reformers and Progressives have deliberately failed to fulfil their pledges in regard to this ambulance question, that the two parties should now accuse each other of being responsible, when both are equally to blame for their neglect of a plain duty. The truth is that politics ought to be excluded altogether from Municipal and County Council business.

In this connection we regret to see under "County Council Notes" in the *Morning Post* of the 17th inst. one headed "Ambulances in London," in which an attempt is made to excuse the Municipal Reformers for their neglect on the ground that they rightly wish to shelve the question so far as the London County Council is concerned, and hand it over to other authorities for economical reasons. This contention, we beg leave to say, plainly, is sheer nonsense. It is County Council work; the County Council already possesses adequate powers, and the cost of an efficient ambulance service has been ascertained to be small to the vanishing point for such a body, for the total cost is estimated at only £15,000 a year, whilst a penny rate now yields in London, in round figures, £180,000 a year. On the other hand, the *Westminster Gazette* of the 14th inst. puts all the blame of the neglect of this question by the County Council upon the Municipal Reformers. These facts are a striking proof that politics in this country are being degraded every hour, and that at the present time it is difficult, if not impossible, to find evidence which warrants the community in believing that a party politician, whatever his colour, has at the bottom a spark of sincere desire to use his influence to safeguard and uphold the interests of the people. We want in this country in public affairs at the present time earnest men of high principle who put the public interest first, and shape their actions and give their votes on no other principle but this.

